# An Advance Step for Celluloid

**Published:** 1874-09

**Authors:** 


### AN ADVANCE STEP FOR CELLULOID.

Celluloid as a material for plates for artificial teeth has been in the hands of the profession for some time, and the success attained has been various, some attaining usually good results, others failing so frequently, as, on this account, to discard its use. Continually from the beginning to the present time, those most interested have been trying to overcome the difficulties, and insure more nearly general success. These efforts have been directed both to the material and the manner of working it.

Such improvements have been recently made in both these directions as to give great encouragement for its future usefulness.

Different and, it is claimed, much better material is now used in its composition than heretofore. The coloring and strength of the material is certainly an improvement on the former product.

The manner of manipulation has been greatly simplified, rendered much easier, and far less unpleasant, steam is used instead of oil; all the appliances have been improved. As the material is now presented there is no scaling or shrinking from the teeth or pins.

In regard to durability we presume it will be quite satisfactory, and with our present views, we have no hesitancy in saying to all, give it a fair and thorough test.

